# The Effect of Transparent/Black Film and Straw Mulching on Canopy Conductance in Maize

**DOI:** 10.3390/plants14182877

**Published:** 2025-09-16

**Authors:** Shanshan Qin, Yanqun Zhang, Xiyun Jiao, Yan Mo, Shihong Gong, Zhe Gu, Baozhong Zhang

**Affiliations:** 1 College of Agricultural Science and Engineering, Hohai University, Nanjing, 210098, China; qinshanshan20@126.com (S.Q.); xyjiao@hhu.edu.cn (X.J.); zhegu2018@hhu.edu.cn (Z.G.); 2 State Key Laboratory of Water Cycle and Water Security, China Institute of Water Resources and Hydropower Research, Beijing 100038, China; moyan@iwhr.com (Y.M.); gshh@iwhr.com (S.G.); zhangbz@iwhr.com (B.Z.); 3 Department of Irrigation and Drainage, China Institute of Water Resources and Hydropower Research, Beijing 100038, China

**Keywords:** film mulching, straw mulching, canopy conductance, microenvironment, vapor pressure deficit

## Abstract

Canopy conductance (*G_c_*) is an important biological constant for quantifying the water vapor flux at the canopy-atmosphere interface, reflecting the coupling strength between crop transpiration and microclimate. To elucidate how mulching modulates *G_c_* dynamics under varying environments, we measured the transpiration of maize based on thermal equilibrium method from 2020 and 2021, synchronously recording solar radiation (*R_s_*), temperature (T), relative humidity (RH), and vapor pressure deficit (VPD) under no-mulching (NM), transparent film (TFM), black film (BM), and straw mulching (SM) treatments in the North China Plain. The results showed that in the near-surface microenvironment, at early stages (seedling-jointing), compared to the NM treatment, TFM and BM treatments unexpectedly reduced temperature by 0.1–1.1% while increasing humidity by 0.2–4.0%, lowering VPD by 0.7–15.5%, contradicting presumed warming effects. During tasseling-filling stages, both plastic films elevated temperature by 3.5–5.2%, decreased humidity by 5.2–6.9%, and sharply increased VPD by 23.4–27.6%, inducing heat-VPD coupling stress. Throughout the entire growth period, SM treatment resulted in an initial increase followed by a decrease in temperature, but the decrease in humidity and increase in VPD occurred earlier and smoothly compared to film mulching treatment in the near-surface microenvironment. All treatments increased average temperature but decreased average humidity in the near-ground microenvironment throughout growth stages, ultimately leading to an increase in average VPD. In addition, all treatments increased *G_c_* at noon by 10.3–81.2%. Under different solar radiation conditions, TFM, BM, and SM treatments increased the reference conductance (*G_cR_*) but did not always increase *G_c_* sensitivity to VPD (m). We propose a specific mulching strategy: Using black or transparent plastic film mulching in arid/cold regions and straw mulching in high-temperature and drought-prone/rain-fed agricultural areas can reconcile the trade-off between microclimate optimization and physiological adaptation, advancing precision water management in arid-prone croplands.

## 1. Introduction

Canopy conductance (*G_c_*) is a key regulatory factor for carbon, water, and heat exchange between vegetation and the atmosphere. Reliable and reasonable estimation of canopy conductance is of great significance for quantifying the material and energy exchange of land surface evapotranspiration [1]. Accurate determination of *G_c_* is important preparatory work for analyzing plant canopy simulation. Some researchers obtained *G_c_* through scale conversion of stomatal conductance measured using various porosimeters [2]. There are also studies that often use noon data to quantify and simulate the influencing factors of *G_c_*. However, these methods not only have significant errors but also make it difficult to continuously measure *G_c_* for a long time. Currently, *G_c_* is generally obtained by the Sapflow method and calculating it using the Penman–Monteith method, which is a commonly used method for obtaining *G_c_*. This method can be used to obtain long-term *G_c_* in a relatively continuous and stable manner [3].

There are five main external environmental factors that affect the opening and closing of stomata or the size of stomatal resistance: solar radiation, air saturation, temperature, CO_2_ concentration in the air, and soil water supply conditions. The effect of small changes in CO_2_ on stomatal conductance is negligible, so it is not necessary to consider the influence of CO_2_ concentration in the air on stomatal conductance during analysis [4]. In research on crop *G_c_*, Jarvis-type multiplication models are commonly used. The Jarvis model typically associates changes in canopy conductance with many meteorological variables, such as temperature (T), saturated vapor pressure deficit (VPD), and solar radiation (*R_s_*). When soil moisture is sufficient, radiation and saturated water vapor pressure difference are the most critical factors determining leaf stomatal conductance [5,6]. Some researchers have studied the effect of temperature rise on soybean transpiration rate and canopy conductance in a temperature gradient chamber under dry and humid conditions. The results indicate that the decrease in canopy conductivity under high-temperature treatment is more likely due to an increase in VPD rather than an increase in temperature itself. There is a non-linear relationship between temperature and *G*_c_. Under drought conditions, the overall response trend to temperature and VPD changes is similar to that under abundant water conditions [7]. However, their quantitative relationship is not clear. Therefore, the response of canopy conductance to temperature and VPD needs further exploration.

Although there has been extensive research on *G_c_* [8], there is relatively little research on the ecosystem of maize in the field. Maize is an important grain, economic feed crop, and industrial raw material in China [9]. In recent years, there have been some studies on the transpiration rate and *G_c_* of maize [10,11]. Previous studies have shown that transparent film, black film, and straw mulching can increase soil moisture and improve the field environment [11,12], but there are few studies that specifically elucidate the relationship between *G_c_* of maize under different mulching conditions and external meteorological variables. Therefore, the daily and seasonal trends of maize *G_c_* under different mulching conditions need further study, and the relationship between the maize external environment (VPD and *R_s_*) and *G_c_* under mulching conditions needs to be clarified, which is beneficial for further clarifying the synergistic relationship between microclimate optimization and physiological adaptation.

Field experiments were conducted in an experimental station in Beijing, China, Water Resources and Hydropower Research Institute, from 2020 to 2021. The specific objectives of this study are as follows: (Ⅰ) to analyze the canopy conductivity dynamics through the entire growth stage in maize fields under no-mulching, transparent film, black film and straw mulching; (Ⅱ) to investigate the effect of meteorological factors on canopy conductivity; (Ⅲ) to quantify the relationship between canopy conductivity and meteorological factors under different mulching treatments.

## 2. Materials and Methods

### 2.1. Research Site and Experimental Design

**Figure 1:**
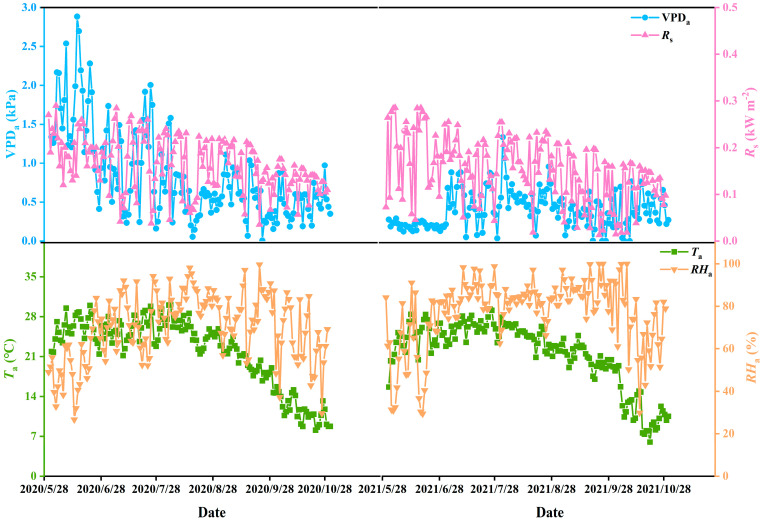
Changes in atmospheric environment during field experiments.

This experiment was conducted at the Daxing Experimental Station of China Water Resources and Hydropower Research Institute in Daxing District, Beijing (39°39′ N, 116°15′ E). The local climate is a typical semi-arid continental monsoon climate, with an average annual rainfall of 540 mm. The soil of the 0–100 cm layer in the experimental field is loam. The average field capacity (FC) and soil bulk density of the 0–100 cm soil layer are 0.306 cm^3^/cm^3^ and 1.41 g/cm^3^, respectively. The field experiment was conducted between June and October from 2020 to 2021. In 2020, the sowing and harvesting times were 30 June and 17 October, while those for 2021 were 26 June and 7 October. The environmental variables during the growing season exhibited significant seasonal and interannual variations (Figure 1). From June to October, the average air temperature (*T_a_*) and solar radiation (*R_s_*) in the atmospheric environment in 2020 were higher than in 2021. The *T*_a_ in July was higher than in other months. The air relative humidity (*RH_a_*) in 2020 was lower than in 2021, but air vapor pressure deficit (VPD_a_) in 2020 was higher than in 2021. The average VPD_a_, *R_s_*, *T_a_*, and *RH_a_* for both years in the atmospheric environment were 23.34 °C, 80.33%, 0.17 kW m^−2^, and 0.57 kPa.

The summer maize was sown by machine using the conventional wide and narrow row planting mode, with wide row spacing of 80 cm, narrow row spacing of 40 cm, plant spacing of 20 cm, and planting density of 83,300 plants/ha. Taking the non-mulching treatment (NM) as a control, three mulching treatments were set up in the experiment, with three replicates of each treatment, including the transparent film (TFM), black film (BM), and straw (SM). Please refer to Qin et al. (2022) [12] for specific information on the experimental setup. When the soil water content (SWC) of transparent film mulching treatment dropped to 60% FC, all treatments were applied with irrigation. In 2020, due to rainfall after sowing, the seedlings were not irrigated. The total rainfall in 2020 and 2021 was 309.63 mm and 341.88 mm, respectively. The specific water and fertilizer system is shown in the table below (Table 1).

### 2.2. Environmental Data Measurement

Meteorological data such as solar radiation, atmospheric temperature, relative humidity, rainfall, wind speed, and so on can be automatically collected through small weather stations within the experimental station (Monitor Sensors, Caboolture, QLD, Australia). The soil water content was measured using the soil moisture probe and data collector (Decagon Em50 Series, Decagon, Pullman, WA, USA), and can be set to automatically record data every 30 min and download data every 10 days.

The air temperature and relative humidity in the near-surface microenvironment were monitored by an automatic-datalogging sensor (Hobo Pro V2, ONSET, Bourne, MA, USA), which was installed 20 cm above the ground surface for all treatments. The data acquisition interval was once every 30 min.

### 2.3. Transpiration Measurement

From the late jointing stage to the heading stage, four sap flow sensors based on thermal equilibrium theory (flow 32-1K, Dynamax, Houston, TX, USA) were installed in each plot. Sap flow rate (*Q*, g h^−1^) was recorded every 30 min by a CR1000 data logger (Campbell Scientific Inc., North Logan, UT, USA). At the same time, the stem cross-sectional area of plants wrapped in instruments and 20 randomly selected continuous plants at a distance of 20 cm above the ground surface was measured (*A*_si_ and *A*_sa_). Calculate *T*_r-SF_ (mm h^−1^) using the equation:

(1) $$T_{r-SF}=\frac{10\times J_{s}\times A_{sa}}{A\times B}$$

(2) $$J_{s}=\frac{Q}{A_{si}}$$

where *A* and *B* are the plant spacing and row spacing, respectively, cm; *J*_s_ is flux density (g cm^−2^ h^−1^); *A*_sa_ is the cross-sectional area of stem, cm^2^; *Q* is the sap flow rate, g h^−1^; *A*_si_ is the cross-sectional area of the stem wrapped 20 cm above the ground, cm^2^; The accumulated *T*_r-SF_ for 24 h is the daily transpiration of the plot, mm d^−1^.

### 2.4. Canopy Conductance Measurement

Based on the P-M equation formula, the canopy conductance (*G*_c_) was calculated from sap flow data:

(3) $$G_{c}=\frac{\gamma \lambda E_{c}G_{a}}{\Delta \left(R_{n}-G\right)+K_{time}\rho C_{p}VPDG_{a}-\lambda \left(\Delta +\gamma \right)E_{c}}$$

where *G*_c_ is the canopy conductance, m s^−1^; γ is the hygrometer constant, kPa °C^−1^; λ is the latent heat of vaporization of water, MJ kg^−1^; *E*_c_ is the transpiration water consumption, mm h^−1^; *G*_a_ is the aerodynamic conductance, m s^−1^, Δ is the slope of the saturated vapor pressure temperature curve, kPa °C^−1^; *R*_n_ is the net radiation, MJ m^−2^ h^−1^; *G* is the soil heat flux, MJ m^−2^ h^−1^; ρ is the air density, kg m^−3^; *C*_p_ is the specific heat at constant pressure of air, MJ kg^−1^ °C^−1^; VPD is the vapor pressure deficit, kPa; and *K*_time_ is the unit conversion coefficient, for 3600 s h^−1^.

The formula for aerodynamic conductance (*G*_a_) was derived as follows:

(4) $$G_{a}=\frac{k^{2}u}{ln\left(\left(z-d\right)/\left(h_{c}-d\right)\right)ln\left(\left(z-d\right)/z_{0}\right)}$$

(5) $$z_{0}=\left\{\begin{array}{c} {z_{0}}^{\prime }\;+\;0.3h_{c}X^{0.5}\;\;\;\;\;\;0<X<0.2\\ 0.3h_{c}\left(1-d/h_{c}\right)\;\;\;0.2<X<1.5\; \end{array}\right.$$

(6) $$d=1.1ln\left(1+X^{0.25}\right)$$

where *k* is the Kalman constant, 0.40, *z* is the reference height, m, which is the measurement height of wind speed and temperature and humidity; *d* is the zero plane displacement, m; *u* is the horizontal wind speed at the reference height, m s^−1^; and z_0_ is the rough length of momentum transmission, m; *h*_c_ is the crop height, m; z_0_′ is the roughness length of bare land, set as 0.01 m, *X* = cdLAI, and cd is the drag coefficient, set as 0.07 in this study [13,14].

### 2.5. Data Analysis

To avoid the influence of other factors, the boundary line analysis method is used to analyze the relationship between *R_s_*, VPD, and *G*_c_: (1) *G_c_* is divided into five intervals according to *R_s_* and VPD, respectively; (2) Calculate the mean and standard deviation of each subinterval; (3) Remove abnormal data; (4) Choose a number greater than the sum of the mean and standard deviation of the interval; (5) Divide the filtered data into three intervals and calculate the mean of *G_c_*, *R_s_*, and VPD. According to the regression lines of boundary line analysis, a reference conductance (*G*_cR_) was calculated at VPD = 1 kPa. The slope of the fitting line quantified the sensitivity of *G*_c_ to VPD (m). When there is no significant difference in the relationship between two parameters in different treatments, perform regression analysis on all data together. All pictures were drawn using Origin 2021 (OriginLab, Northampton, MA, USA).

## 3. Results

### 3.1. Environmental Parameters

#### 3.1.1. The Near-Surface Microenvironment

**Figure 2:**
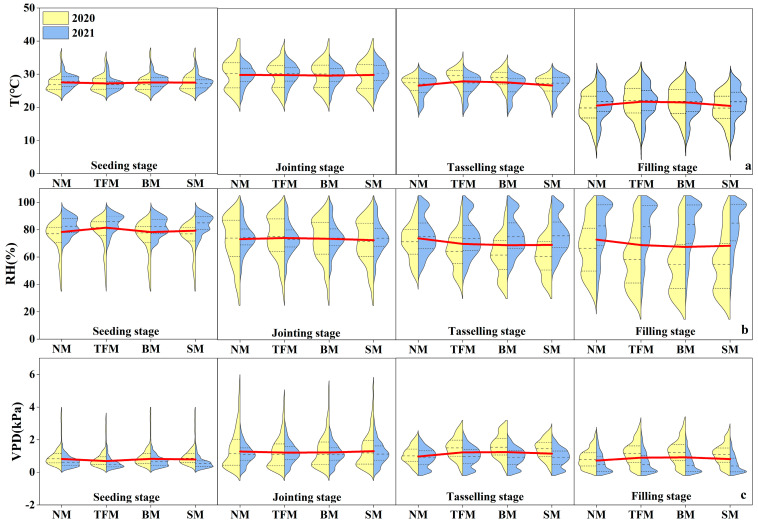
Changes in (**a**) temperature (T), (**b**) relative humidity (RH), and (**c**) vapor pressure deficit (VPD) in field microenvironment under different mulching treatments. NM—no mulching, TFM–transparent film mulching, BM—black film mulching, SM—straw mulching. The red line indicates the average value for both years.

During the two growing seasons, air temperature in the near-surface microenvironment increased from the seeding stage to the jointing stage and started to decrease in the tasseling stage. Under the NM treatment, the mean air temperatures were 27.77 °C, 29.98 °C, 26.82 °C, and 20.84 °C for the seeding, jointing, tasseling, and filling stages, respectively (Figure 2a). The TFM and BM treatments decreased the near-surface temperatures by 0.1–1.1% from the seeding stage to the jointing stage but increased them by 3.5–5.2% from the tasseling to the filling stage. In contrast, the SM treatment resulted in a slight increase (0.1%) from seeding to tasseling stages but a minor decrease (0.6%) at the filling stage. The average near-surface temperature during the growth period under the NM treatment was 25.7 °C, with TFM and BM increasing by 2.1% and 1.6%, and SM decreasing by 0.2%. The near-surface temperature in all treatments was higher than the atmospheric temperature.

During the two growing seasons, the near-surface RH decreased from the seeding stage to the filling stage. The mean near-surface RH at the seeding, jointing, tasseling, and filling stages under the NM treatment was 78.67%, 73.52%, 73.88%, and 73.35%, respectively (Figure 2b). The TFM and BM treatments increased the near-surface RH by 0.2–4.0% from the seeding stage to the jointing stage but decreased by 5.2–6.9% from the tasseling stage. The SM treatment increased the near-surface RH by 0.9% at the seeding stage but decreased by 1.0–6.6% from the jointing to filling stage. The average near-surface RH during the growth period under the NM treatment was 74.6%, with TFM, BM, and SM decreasing by 1.6%, 3.5%, and 3.1%, respectively. The near-surface RH in all treatments was lower than the atmospheric RH.

During the two growing seasons, the near-surface VPD increased from the seeding stage to the jointing stage and started to decrease in the tasseling stage. Mean near-surface VPD at the seeding, jointing, tasseling, and filling stages under the NM treatment were 0.84 kPa, 1.27 kPa, 0.98 kPa, and 0.73 kPa, respectively (Figure 2c). From the seeding to the jointing stage, the TFM and BM treatments decreased the near-surface VPD by 0.7–15.5%, but increased it by 23.4–27.6% from the tasseling stage. The SM treatment decreased the near-surface VPD by 2.4% at the seeding stage but increased by 2.2–17.9% from the jointing to filling stage. The average near-surface VPD during the growth period treated by NM was 0.93 kPa, with TFM, BM, and SM increasing by 6.4%, 10.9%, and 6.3%, respectively. The near-surface VPD in all treatments was higher than the atmospheric VPD.

#### 3.1.2. Soil Water Content

**Figure 3:**
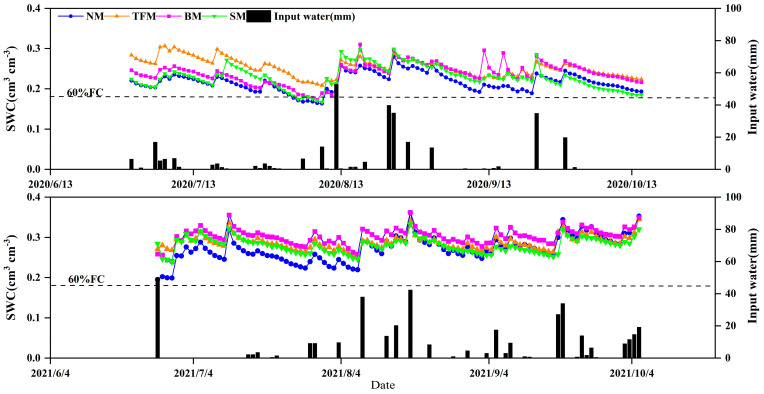
Changes in soil water content (SWC) of maize under different treatments. NM—no mulching, TFM—transparent film mulching, BM—black film mulching, SM—straw mulching. The input water includes rainfall and irrigation. The dashed line refers to 60% field capacity (FC).

The average soil water content (SWC) in the 0–40 cm soil layer during the 2020 growth season in the NM, TFM, BM, and SM treatments ranged from 0.22 cm^3^cm^−3^ to 0.25 cm^3^cm^−3^ (Figure 3). Compared with NM treatment, the TFM, BM, and SM treatments significantly increased SWC by 13.6%, 9.1%, and 4.6%, respectively (*p* < 0.001). In 2021, the SWC in the 0–40 cm soil layer ranged from 0.27 cm^3^ cm^-3^ to 0.30 cm^3^ cm^-3^ (Figure 3). Compared with NM treatment, the TFM, BM, and SM treatments significantly increased SWC by 7.4%, 11.1%, and 3.7%, respectively (*p* < 0.001). In 2020, both NM and SM treatments experienced SWC below 60% field capacity (FC) for 7 days. The BM treatment reduced the days of SWC below 60% FC by only 4 days, while the SWC of the TFM treatment did not fall below 60% FC. In 2021, the SWC of all treatments was higher than 60% FC, and SWC was not a limiting factor for maize growth.

### 3.2. Changes in Canopy Conductance Under Different Mulching Treatments

**Figure 4:**
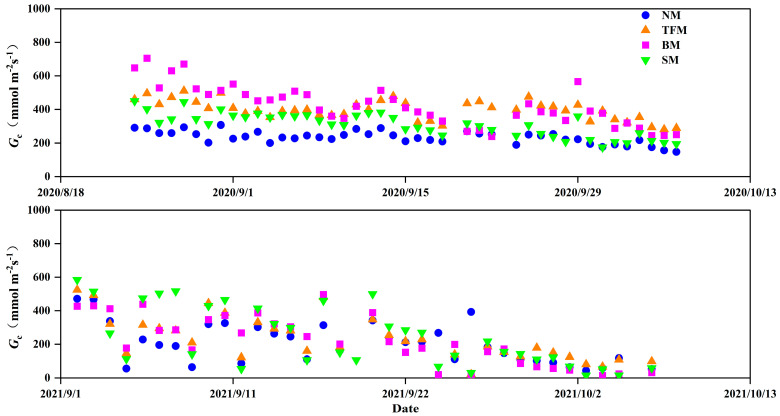
Changes in canopy conductance (*G*_c_) from 11:00 to 13:00 at noon under different mulching treatments. NM—no mulching, TFM—transparent film mulching, BM—black film mulching, SM—straw mulching.

Canopy conductance (*G*_c_) was analyzed between 11:00 and 13:00 during the growing season. The average midday G_c_ of NM, TFM, BM, and SM treatments in 2020 were 233.90, 398.34, 423.78, and 308.56 mmol m^−2^s^−1^, respectively (Figure 4). These represent increases of 70.3%, 81.2%, and 31.9%, respectively, compared to NM treatment. The mean midday *G_c_* of NM, TFM, BM, and SM treatments in 2021 were 202.98, 235.22, 223.97, and 242.34 mmol m^−2^s^−1^, respectively. The midday *G_c_* of TFM and SM treatments increased by 15.9%, 10.3%, and 19.4%, respectively, compared to NM treatment.

### 3.3. Response of Canopy Conductance to Environmental Factors

**Figure 5:**
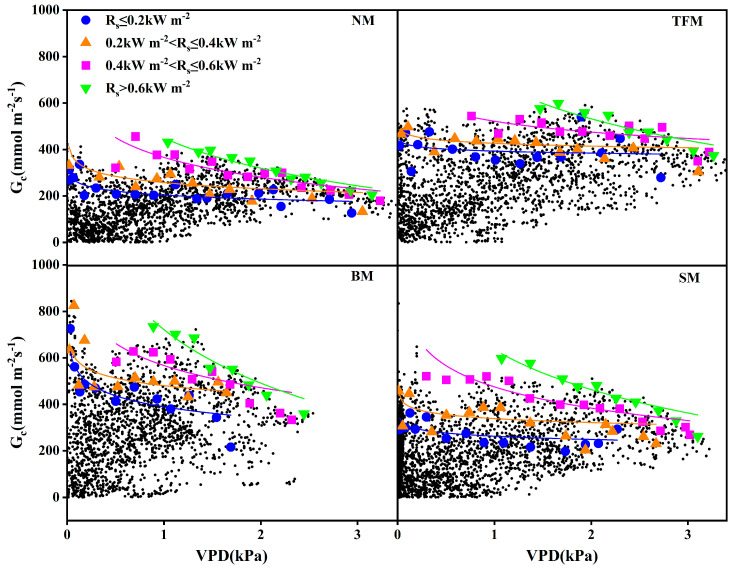
Relationship between vapor pressure deficit (VPD) and canopy conductance (*G*_c_) under different mulching treatments. NM—no mulching, TFM—transparent film mulching, BM—black film mulching, SM—straw mulching. The curves were calculated by the boundary line analysis method.

Boundary-line analysis between VPD and *G_c_* was performed for each treatment after partitioning the data into four consecutive ranges of *R_s_* (Figure 5). The results of regression analysis between VPD and *G_c_* under different mulching treatments are presented in Table 2. It can be shown that compared to NM treatment, all mulching treatments increased the *G_c_*. When VPD < 1 kPa, *G_c_* rapidly declined with the increase in VPD. When VPD = 1 kPa, the *G_c_* among all treatments increased with the increase in *R_s_*. When VPD > 1 kPa, the difference in *G_c_* caused by *R_s_* decreased with the increase in VPD.

**Figure 6:**
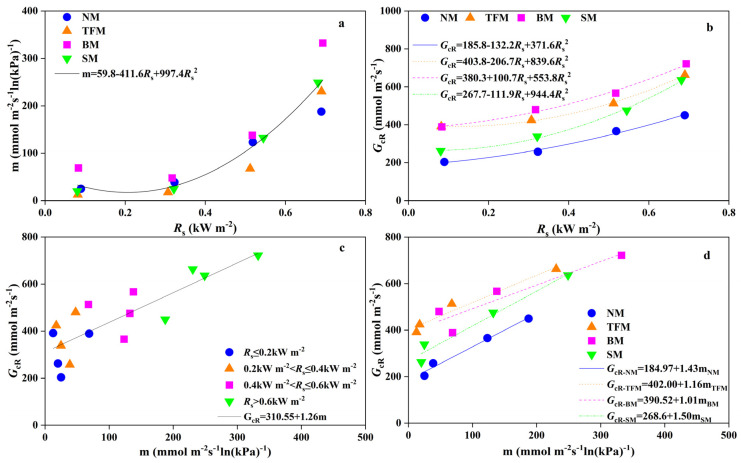
Relationship between (**a**) canopy conductance sensitivity to vapor pressure deficit (m) and solar radiation(*R*_s_), (**b**) reference conductance (*G*_cR_) and *R*_s_, (**c**) *G*_cR_ and m under different *R*_s_ ranges, and (**d**) *G*_cR_ and m under different mulching treatments. NM—no mulching, TFM—transparent film mulching, BM—black film mulching, SM—straw mulching. When there is no significant difference in the relationship between two parameters in different treatments, perform regression analysis on all data together.

**Figure 7:**
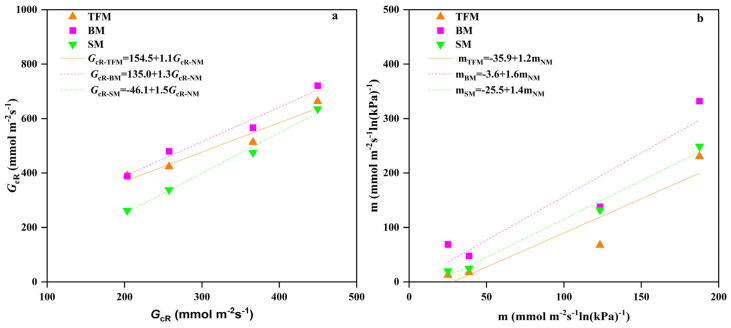
The analysis of (**a**) reference conductance (*G*_cR_) and (**b**) canopy conductance sensitivity to vapor pressure deficit (m) under different mulching treatments. NM—no mulching, TFM—transparent film mulching, BM—black film mulching, SM—straw mulching. The lines were obtained through regression analysis.

From boundary-line analysis, the reference conductance (*G_cR_*) and *G_c_* sensitivity to VPD (m) were obtained by processing each *R_s_* interval with different mulching treatments. The m and *G_cR_* showed a decrease followed by an increase with the rising *R_s_* (Figure 6a,b). Under different *R*_s_ conditions, TFM, BM, and SM treatments increased the *G_cR_*, but did not always increase m. There was no significant correlation between m and *G_cR_* under different solar radiation conditions. A robust linear correlation between *G_cR_* and m (R^2^ = 0.80–0.97) was established across treatments (Figure 6c,d). Compared to the NM treatment, the intercept of the fitting line between *G_cR_* and m under the TFM, BM, and SM treatments increased by 117.3%, 110.8%, and 45.2%, respectively. The *G_cR_* and m of TFM, BM, and SM treatments showed a linear correlation with NM treatment (Figure 7a,b).

## 4. Discussion

### 4.1. Differences in Environmental Parameters Under Different Mulching Treatments

The air temperature is mainly determined by the solar radiation, turbulent exchange, and evapotranspiration within the crop population [15]. Researchers in Gansu investigated the air temperature, humidity, and wheat yield in plastic film-covered wheat fields. The results showed that after covering wheat with plastic film, the temperature and relative humidity within the crop population increased, and the increase in yield was more significant [16]. In our study, the near-ground temperature under the transparent film and black film mulching treatment was lower than that in the no-mulching treatment in the early stage of growth, but higher in the late stage of growth. The possible reason was that in the early stage of growth, the leaf area index is relatively small. When sunlight shines directly on the ground, the bare ground can absorb most of the energy to increase the temperature, while the film reflects some of the energy. With the growth and development of crops and the increase in leaf area index, the heat reflected by the film could be reused by the bottom leaves [17]. The effect of straw mulching on near-ground air temperature is opposite to that of film mulching, which makes it possible that straw mulching has a stronger ability to alter turbulent heat exchange, increase evaporation heat consumption, and increase soil heat flux, ultimately reducing near-ground air temperature more than film mulching [18].

The relative humidity of the air in crop populations is mainly determined by air temperature, field evapotranspiration, and turbulence exchange intensity. There are studies showing that the relative humidity at 30 cm above the ground surface was plastic film mulching < straw mulching < bare land [19]. The film mulching and straw mulching treatments reduce the upward water evaporation from the soil due to the film blocking some of the soil evaporation. This is consistent with our research findings that the soil water content under film and straw mulching treatments is significantly higher than that under no mulching treatment (Figure 3). In addition, we also found that the relative humidity of the lower layer under the transparent film, black film, and straw mulching treatments was higher than that under the no-mulching treatment in the initial stage of crop growth, while the opposite was true in the late stage of crop growth. The possible reason is that in the initial stage of crop growth, plants grow relatively short, and the soil surface is the surface that receives solar radiation for heat exchange. During the day, the water vapor pressure within the crop population gradually increases as the height decreases from the ground level layer downward. A high-humidity microenvironment is formed under the film and straw mulching, where water vapor slowly escapes through plant pores or membrane damage, providing a stable source of water vapor for near-surface air with high humidity [20]. The evaporation under the no mulching treatment is greater, and it directly and quickly dissipates into the higher atmosphere, making it difficult to maintain high humidity near the surface. During the vigorous period of crop growth and development, crop transpiration dominates [17]. The crop canopy is closed, and the evaporated water is blocked below the canopy, while the soil covered with film evaporates less than the uncovered treatment. Therefore, the relative humidity of the lower layer under the film mulching treatment is lower than that under the no mulching treatment. However, the humidity reduction of straw mulching treatment was earlier than that of film mulching treatments, which may be due to the strong cooling effect under the straw mulching. This may be achieved by delaying crop growth, directly absorbing water vapor, resulting in an earlier decrease in near-surface air humidity under straw mulching than under film mulching. Film mulching mainly promotes crop growth through strong warming effects, shifting the dominant factor of microclimate in farmland from evaporation to transpiration, thereby significantly reducing near-surface air humidity later than straw mulching.

The relationship between soil moisture and saturated vapor pressure deficit (VPD) in the field atmosphere is one of the core aspects of agricultural meteorology and crop physiology [21]. They are interconnected and influenced by the Soil Plant Atmosphere Continuum (SPAC). When the soil moisture is sufficient, both soil evaporation and crop transpiration are strong, the ET amount is large, and the amount of water vapor transported to the atmosphere is high, increasing air humidity and thus reducing VPD. A high VPD environment (atmospheric drought) can also exacerbate soil water consumption and crop water stress in reverse. Studies have shown that reducing VPD appropriately can effectively maintain water balance and promote plant development [22]. However, we found that throughout the entire growth period, the rainfall in 2021 was higher than in 2020, resulting in higher atmospheric humidity and lower atmospheric VPD in 2021. Compared to the no mulching treatment, VPD in the near-surface microenvironment was reduced first under TFM, BM, and SM treatments but increased later. This phenomenon is driven by the phased regulation of energy allocation, water transport, and crop physiology through mulching measures. The film mulching and straw mulching increased soil water content. During the early stages, the plant canopy was not closed, and a high-humidity microenvironment was provided by a stable source of water vapor under the film and straw mulching, resulting in a significant decrease in VPD. In the later stage of growth, the LAI reached its maximum, and the plant canopy was closed. The near-surface VPD is mainly influenced by water vapor below the canopy. The main source of water vapor below the canopy is soil evaporation, while the evaporation of soil covered with film and straw is lower than that of the uncovered treatment, resulting in lower humidity and increased VPD.

### 4.2. Effects of Different Mulching Treatments on G_c_

The physiological and ecological processes of the vegetation canopy can be simulated to explore the interrelationships between environmental factors and vegetation canopy, which can provide effective methods for simulating ecosystem productivity, the interaction between terrestrial ecosystem processes and climate, and predicting ecological environment changes at a larger scale. On the annual timescale, we found that the growing seasons in 2020 were drier compared with 2021. The *G_c_* in the growing season of 2020 was significantly higher than that in the relatively wet 2021. In addition, *G_c_* in the microenvironment under TFM and BM treatments was higher than in the NM treatment, which may be because the TFM and BM treatments increased the soil water content and VPD in the microenvironment at the tasseling and filling stages. The high soil water availability and transpirational pull and high atmospheric evaporative demand result in a significant increase in canopy transpiration and *G_c_* [23,24,25].

The variation process of canopy stomatal conductance degree based on sap flow calculation shows obvious regular fluctuations with environmental factors such as solar radiation, vapor pressure deficit, and atmospheric temperature, but the process and pattern of changes are not synchronized. Saturated vapor pressure difference and photosynthetically active radiation (PAR), as environmental factors affecting plant transpiration, are the two most critical factors affecting it on a short time scale (hourly, day-night) [26,27]. Studies have shown that PAR has a positive correlation with stomatal conductance in the canopy of *Platycodon grandiflorus* within the range of 0–400 μmol m^−2^ s^−1^. Beyond this value, sensitivity to PAR is relatively low. There is a threshold for the effect of photosynthetically active radiation on canopy stomatal conductance. Within this threshold range, PAR has a greater driving force on canopy stomatal conductance, while beyond it, the effect is weaker [28]. The influence of canopy conductance on evapotranspiration showed a threshold effect [29]. However, some studies have shown that stomatal conductance in the canopy of forests is linearly positively correlated with photosynthetically active radiation, and no threshold phenomenon has been observed [30]. A study conducted on canopy stomatal conductance of *Acacia matsuta* found that its response to PAR increased in a hyperbolic function [31]. We found that with the increase in solar radiation, the difference between canopy conductance gradually decreases. It could be seen that there was a threshold phenomenon.

Previous research showed that soil moisture can influence the response of canopy transpiration and conductance to meteorological factors [23,32,33,34], but the effect of atmospheric drought on water use has been ignored. Atmospheric drought and soil drought together affect canopy transpiration [35]. The variability of *G_c_* was empirically attributed to VPD and soil water supply [36]. The transpiration could be increased with the increase in atmospheric evaporative demand when soil water was sufficient [33]. The high soil water content and low VPD were beneficial to leaf stomatal opening; the *G_c_* was relatively high [37]. Under atmospheric drought, leaf stomata were closed early to reduce *G_c_*. Under soil drought, VPD could still promote transpiration due to low atmospheric evaporative demand. VPD has a low inhibitory effect on *G_c_* [23]. Wang et al. (2017) also found that when VPD < 1.5 kPa, the canopy conductance of *Artemisia annua* showed a positive linear correlation with VPD [38]. To avoid interference from other factors, perform boundary line analysis on *G_c_* and VPD. However, our studies have shown that there was a negative logarithmic relationship between canopy stomatal conductance and saturated water vapor pressure difference. The higher the VPD, the lower the stomatal conductance, which was similar to Zhao et al. (2006), who found plants close some stomata to prevent excessive water evaporation and ensure water storage in their bodies under high VPD [31]. Compared to the NM treatment, the TFM, BM, and SM treatments increased *G_cR_* and m. Under different *R_s_* conditions, all treatments increased the reference conductance *G_cR_*, as well as the intercept of the fitting line between *G_cR_* and m, but did not always increase m. This suggested that *G_c_* gains may mainly be due to the root hydraulic improvements (e.g., enhanced water uptake under film mulching) rather than stomatal adjustments [39].

In conclusion, the VPD difference of crops at different growth stages could have a certain impact on crop canopy conductance. The use of films and other mulching measures can increase canopy conductivity. However, the impact of different surface mulching measures on the field microenvironment varies at different stages of growth. According to our study, plastic film mulching for early growth exploited its VPD-lowering effect in the near-surface microenvironment, and straw mulching at tasseling could mitigate late-stage VPD spikes. This may be related to the effects of film warming and straw cooling [40,41]. Therefore, the transparent film, black film, and straw mulching could all increase *G*_c_, and carbon, water, and heat exchange between vegetation and the atmosphere can be better regulated under these mulching conditions. In arid/cold regions, it is recommended to use black or white plastic film mulching throughout the entire growth period. In high-temperature and drought-prone areas/rain-fed agricultural areas, it is recommended to use straw mulching throughout the entire growth period.

## 5. Conclusions

This study uncovers a paradoxical microclimate trajectory under plastic film mulching: initial cooling and VPD reduction in the near-surface microenvironment during early growth stages shift to significant warming and VPD amplification post-tasseling. The air temperature in the near-surface microenvironment did not increase at seedling-jointing stages under transparent/black films, which challenges conventional expectations of mulching-induced warming, attributable to radiation reflection (transparent film) and suppressed sensible heat flux (both films). Conversely, the 4% temperature rise and 25% VPD surge during grain filling arise from soil heat accumulation and enhanced turbulent diffusion of vapor under high LAI conditions.

Straw mulching uniquely delivered stable VPD suppression throughout the season but provided lesser canopy conductance (*G_c_*) gains than plastic films, reflecting a trade-off between microclimate stability and physiological stimulation. The VPD-*G_c_* disconnection under straw implies its effects are mediated via soil moisture conservation rather than canopy microclimate modification.

Critically, all mulching (films/straw) treatments elevated the *G_c_* by 10.3–81.2% compared to no-mulching treatment, confirming their role in improving hydraulic efficiency. However, under different *R_s_* conditions, all treatments increased the reference conductance (*G_cR_*) but did not always increase *G_c_* sensitivity to VPD (m). This finding indicates that mulching enhances water transport capacity without modifying stomatal regulatory behavior. This suggested that *G_c_* gains stem primarily from root hydraulic improvements (e.g., enhanced water uptake under film mulches) rather than stomatal adjustments.

We thought of a stage-specific mulching strategy: In arid/cold regions, it is recommended to use black or white plastic film covering throughout the entire growth period. In high-temperature and drought-prone areas/rain-fed agricultural areas, it is recommended to use straw mulching throughout the entire growth period. This reconciles the trade-off between microclimate optimization and physiological adaptation, advancing precision water management in arid-prone croplands.

## Figures and Tables

**Table 1:** Irrigation and fertilizer management for 2020 and 2021.

Year		Reproductive Period	Date	Irrigation Amount (mm)	Fertilization Amount (kg ha^−1^)		
					N	P_2_O_5_	K_2_O
2020	1	Jointing stage	8/1	5	156	108	135
	2	Heading stage	8/16	5	48	27	
	3	Filling stage	9/13	5	36		
	Total			15	240	135	135
2021	1	Seedling stage	6/27	50	156	108	135
	2	Jointing stage	8/2	5	156	108	135
	3	Heading stage	8/22	5	84	27	
	Total			60	240	135	135

**Table 2:** The regression equations between vapor pressure deficit (VPD) and canopy conductance (*G*_c_) under different mulching treatments. NM—no mulching, TFM—transparent film mulching, BM—black film mulching, SM—straw mulching.

Treatments	*R*_s_ ≤ 0.2 kW m^−2^	0.2 < *R*_s_ ≤ 0.4 kW m^−2^	0.4 < *R*_s_ ≤ 0.6 kW m^−2^	*R*_s_ > 0.6 kW m^−2^
NM	*G*_c_ = 203.4 − 25.0 × ln(VPD)	*G*_c_ = 257.4 − 38.7 × ln(VPD)	*G*_c_ = 365.8 − 123.3 × ln(VPD)	*G*_c_ = 449.4 − 187.6 × ln(VPD)
TFM	*G*_c_ = 391.1 − 12.6 × ln(VPD)	*G*_c_ = 424.0 − 17.4 × ln(VPD)	*G*_c_ = 512.9 − 67.6 × ln(VPD)	*G*_c_ = 662.9 − 230.4 × ln(VPD)
BM	*G*_c_ = 389.1 − 68.8 × ln(VPD)	*G*_c_ = 480.0 − 47.7 × ln(VPD)	*G*_c_ = 566.9 − 137.9 × ln(VPD)	*G*_c_ = 721.5 − 332.4 × ln(VPD)
SM	*G*_c_ = 262.2 − 20.1 × ln(VPD)	*G*_c_ = 338.2 − 24.8 × ln(VPD)	*G*_c_ = 475 − 132.2 × ln(VPD)	*G*_c_ = 635.8 − 248.9 × ln(VPD)

## Data Availability

Data will be made available on request. The data are not publicly available due to ongoing research.
